# To Take a Risk or Not? The Effect of Perceived Scarcity on Risky Choices

**DOI:** 10.3390/bs13090743

**Published:** 2023-09-06

**Authors:** Shujing Liang, Ping Fan, Guangyong Yang

**Affiliations:** School of Management, Guizhou University, Guiyang 550025, China; gs.pfan21@gzu.edu.cn (P.F.); gs.gyyang22@gzu.edu.cn (G.Y.)

**Keywords:** perceived scarcity, decision making, risk taking, risk averse, risky choices

## Abstract

Studies suggest that resource scarcity leads to risky behaviors. From a cognitive perspective, a scarcity mindset affects the decision-making process. Does perceived scarcity therefore affect risk taking when making decisions? This study (N = 213) was conducted in western China to examine the effect of perceived scarcity on risky choices. Our results revealed that participants in the scarcity condition tended to be more risk averse than participants in the control condition when making a risky decision. Perceived scarcity increased the probability of choosing the safe option that offered a sure gain. The effect of psychological variables (emotion, risk attitude, personality, impulsivity, self-control and ego depletion) on risky choices was also tested. Risk attitude, urgency in impulsivity, and deliberate action in self-control also influence risky choices.

## 1. Introduction

Scarcity (e.g., money scarcity and time scarcity) can happen to anyone. Perceived scarcity occurs when people feel they have less than they need or they want [1]. Perceived scarcity refers to individual subjective feelings of scarcity with regard to certain tangible resources or intangible resources [2]. Mullainathan and Shafir (2014) proposed the Scarcity Mindset Theory, which argues that when faced with resource scarcity, people focus on the immediate issue of resources shortage and ignore other relevant information [1,3]. Firstly, unsatisfied needs caused by scarcity capture individual attention in the scarcity condition. This trade-off thinking may cause irrational decision making. Secondly, proactive interference caused by scarcity leads to tunnelling [1]. Tunnelling is a negative consequence of a scarcity mindset: the brain will ignore useful information while blocking interferences and temptations [1]. A series of laboratory and field experiments has suggested that a scarcity mindset influences the decision-making processes.

The impacts of a scarcity mindset on decision-making processes include decreased cognitive functions, negative behaviors and irrational decisions. These effects of scarcity perception on decision making have been identified in previous research. Huijsmans et al. (2019) found that individuals with scarcity perception showed increased activity in the orbitofrontal cortex, and this often affected valuation processes and decreased activity in the dorsolateral prefrontal cortex, which is associated with goal-directed choices [4]. Williams et al. (2016) found that a scarcity heuristic affected reward evaluation processes, which was reflected within the human medial-frontal cortex [5]. According to existing studies, scarcity perception affects decision making processes (e.g., information processing, outcome valuation) and leads to irrational decisions; it also suggests that perceived scarcity affects the decision-making process, particularly with regard to risky decisions. Whether perceived scarcity increases risk taking when making risky decisions has yet to be determined. The goal of this study is to examine the effect of perceived scarcity on risky choices.

According to Life History Theory [6], people living in an environment with resource scarcity will adopt faster strategies. Faster strategies include taking more risks, displaying more aggression and having a preference for immediate benefits [7]. For slower strategies, the reverse is true. When faced with risky choices, people who have adopted faster strategies may show a different risk-taking propensity. Some will tend to be more risk-seeking in pursuit of greater rewards. Others may be more short-sighted, seeking immediate gratification [8]. Risk Sensitivity Theory argues that when the caloric energy budget declines, animals begin risk-seeking and are more inclined to choose a high-risk/high-reward foraging option [9]. Humans also conformed to predictions of Risk Sensitivity Theory [10,11,12,13]: people shift from being risk averse to risk seeking in pursuit of scarce resources and satisfying perceived needs [14,15]. Humans have an instinct to satisfy their needs [16]. Realizing scarcity leads people to try to obtain more to cover the shortage of resources [13]. Thus, we could assume that when making risky choices, people tend to be more risk-seeking in pursuit of greater rewards. However, when we consider risky choices compared with sure choices, some people may choose the safe option to obtain more resources at lower risks. Therefore, the hypothesis that perceived scarcity induces risk aversion was proposed. We conducted an experiment in Chongqing City in western China to investigate whether perceived scarcity increases risk aversion when making risky choices with a safe choice.

The main difference between the present study and previous studies is that perceived scarcity is a subjective feeling and it can be induced by both absolute scarcity and relative scarcity. Most existing studies have focused on the effect of absolute scarcity and paid close attention to the impacts of money scarcity (e.g., poverty) on risk taking.

## 2. Methods and Materials

This study was designed as a between-subjects experiment. According to existing studies, emotion [16], sensation seeking [17], personality [17], impulsivity [18], and self-control all influence risk taking [19]. Thus, the psychological variables mentioned above were measured to control the impacts of individual differences on risk taking in the present study.

### 2.1. Method

#### 2.1.1. Participants

The sample size was calculated with G*Power 3.1 in the present study. A priori analysis suggested that at least 128 participants would be sufficient to detect a medium-size effect (effect size = 0.25) with power of 0.8 at the level of 0.05. We recruited 213 undergraduates aged 17 to 21 (*M*_age_ = 18.64, *SD* = 0.75; 59 males, 27.7%), recruited from a university in Chongqing City; the sample size was sufficient to detect a medium-sized effect (effect size = 0.25) with a power of 0.8 at the level of 0.05. None of the participants had previously taken part in a similar experiment before. Each participant received a WeChat red envelope containing a random amount of money between CNY 2.00 (USD 0.29) and CNY 10.00 (USD 1.43).

The study was conducted in accordance with the Declaration of Helsinki, and approved by the Ethics Committee of Guizhou University. Informed consent was obtained from all subjects involved in the study.

#### 2.1.2. Procedure

Participants were recruited through the Dean’s Office and were gathered in a computer classroom. They completed the experiment via filling out an offline questionnaire and an online choice task via an online research platform in China (https://www.wjx.cn/ (accessed on 12 August 2023)). Participants were told that they would take a test pertaining to behavioral tendencies that had been organized by the Dean’s Office. Firstly, participants were asked to report their emotional state, risk attitude, personality, impulsivity, and self-control. Secondly, participants were randomly assigned to the scarcity condition (N = 119) or the control condition (N = 94). To induce perceived scarcity, participants in the scarcity condition were asked to recall memories of experiencing resource scarcity. Participants in the control condition were asked to recall things they did during the last two weeks. Thirdly, participants were asked to complete a four-question questionnaire to examine the manipulating effect. Fourthly, participants were asked to choose how they preferred to receive their reward after completing the test. Each alternative, with the same expected value, was presented as the amount of money and its probability. After the experiment, participants were told the true purpose and the manipulating process of the study.

### 2.2. Manipulation and Measures

Manipulation of Scarcity. An episodic recall task [20] was adopted to manipulate perceived scarcity. During the task, participants in the scarcity condition were asked to describe three or four occasions when they felt like “resources were scarce” or they “did not have enough of something”. Then, they were required to pick two of these experiences and write both down in detail to explain what was lacking and what they experienced. Participants in the control group were asked to describe three or four events that happened in the last two weeks, then pick two of the events and write them down in detail. The tasks were translated from English into Chinese and translated back by different professional researchers to ensure their accuracy and feasibility. According to [20], four questions (i.e., “My resources are scarce”, “I do not have enough resources”, “I need to protect the resources” and “I need to acquire enough resources”) were used to test if the manipulation was successful. The responses were based on a 7-point Likert scale (1 = *strongly disagree*, 7 = *strongly agree*). Considering the language differences, after these questions had been translated into Chinese, the pretest (112 participants) was conducted. The pretest showed good internal consistency for these questions (*Cronbach’s α* = 0.704). The descriptive statistics for the scarcity perception are shown in Table 1.

*Measure of Risky Choice.* The choice task, adapted from the work of Mishra et al. (2015), was adopted [21]. In the choice task, participants were asked to choose how to receive their reward after the experiment. Participants were told the following: “In order to thank you for your participation and support, our research team will provide you with the reward. The following are the ways that you can get the reward. Please indicate which option you prefer, and you will have chance to get money associated with your decision. Please make your decisions as honestly as possible.” As is shown in Table 2, one safe option and six risky options with equal expected values were presented in a random order.

Measure of Emotional State. The Positive Affect and Negative Affect Scale (PANAS) was adopted to measure emotion [22]. The PANAS consists of 20 descriptions of different emotional states. Ten of the items are positive emotions and the other ten items are negative emotions. Each emotion is measured using a 5-point Likert scale (1 = *very slightly or not at all*, 5 = *extremely*). The Chinese version of PANAS, translated and revised by Huang et al. (2003), was used in this study [23]. The pretest results indicate that the PANAS has good internal consistency; the *Cronbach’s α* of the positive emotion scale and negative emotion scale was 0.873 and 0.834, respectively. The descriptive statistics for the emotional state are shown in Table 1.

Measure of Risk Attitude. The General Risk Propensity Scale (GRiPS) was adopted to measure risk attitude [24]. The GRiPS consists of 8 statements of risk attitude (e.g., “Taking risks makes life more fun.”, “I enjoy taking risks in most aspects of my life.”). Participants were asked to indicate the degree to which they agreed or disagreed with the statements on a 7-point scale (1 = *strongly disagree*, 7 = *strongly agree*). The descriptive statistics for risk attitude are shown in Table 1.

Measure of Personality. The Ten-Item Personality Inventory (TIPI), which is a very brief measure of Big-Five personality dimensions, was adopted due to its simplicity [25]. The TIPI comprises five items used to measure personality. Each item consists of two descriptions of personality traits from five dimensions, including extroversion (i.e., “Extraverted, enthusiastic” and “Reserved, quiet”), agreeableness (i.e., “Critical, quarrelsome”, “Sympathetic, warm”), conscientiousness (i.e., “Dependable, self-discipline”, “Disorganized, careless”), emotional stability (i.e., “Anxious, easily upset”, “Calm, emotional stable”), and openness to experience (i.e., “Open to new experiences, complex”, “Conventional, uncreative”). Participants were asked to indicate the extent to which they agreed or disagreed with the statements on a 7-point scale (1 = *disagree strongly*, 7 = *agree strongly*). The descriptive statistics for personality are shown in Table 1.

Measure of Impulsivity. The UPPS impulsive behavior scale (UPPS) was adopted to measure impulsivity [26]. The UPPS contains four subscales (45 items), including Urgency (12 items; e.g., “Sometimes I do things on impulse that I later regret”), Premeditation (11 items; e.g., “I usually think carefully before doing anything”), Perseverance (10 items; e.g., “I generally like to see things through to the end”), and Sensation Seeking (12 items; e.g., “I would enjoy fast driving”). Participants were asked to indicate the degree to which they agree or disagree with the statements on a 7-point scale (1 = *disagree strongly*, 7 = *agree strongly*). The descriptive statistics for impulsivity are shown in Table 1.

Measure of Self-Control. Tangney’s Self-Control Scale (SCS) was used to measure self-control [27]. The Chinese version of SCS that was translated and revised by Unger, Bi, Xiao, and Ybarra (2016) was adopted in the present study [28]. The Chinese SCS contains 36 items and reflects 5 factors, including general ability for self-discipline (11 items), deliberate action (10 items), health habits (5 items), work ethics (5 items), and reliability (5 items). Participants were asked to indicate the extent to which they agreed or disagreed with the statements on a 7-point scale (1 = *disagree strongly*, 7 = *agree strongly*). The descriptive statistics for self-control are shown in Table 1.

According to Dou et al. (2014), ego depletion promotes risk-taking behavior [19]. In a study by Dou et al., in a state of high self-control resources depletion, people who drank beverages containing glucose engaged in less risky behavior than those drinking the beverage without glucose. This suggests that supplementing physiological energy could effectively mitigate the negative effect of self-control resource depletion on risk-taking behavior [19]. Therefore, to control the impacts of ego depletion that resulted from the lack of physiological energy supplementation in our experiment, we collected participants’ information to identify any hypoglycemia diagnoses or self-reported hypoglycemic symptoms. In addition, participants were offered cakes and lollipops from the same brand. Participants were asked if they had confirmed hypoglycemia before the experiment, and after the experiment they were asked if they had experienced hypoglycemic-like symptoms during the experiment or after the experiment.

## 3. Results

### 3.1. The Manipulating Effect

An independent samples t-test indicated that the manipulation of scarcity was validated: participants in the scarcity condition reported a higher average score of scarcity perception (M = 4.45, SD = 0.59) than participants in the control condition (M = 5.33, SD = 0.82), *t*(211) = 8.75, *p* < 0.001, Cohen’s d = 1.27.

### 3.2. The Scarcity Effect

Compared with participants in the control condition (*M* = 0.38, *SD* = 0.33), participants in the scarcity condition reported a lower risk of receiving no money (*M* = 0.29, *SD* = 0.34), *t*(211) = 2.00, *p* = 0.047, Cohen’s d = 0.27. In the scarcity condition, 57 participants (47.9%) selected the safe option, while 28 participants (29.8%) in the control condition selected the safe option, *χ*^2^ (1, *n* = 213) = 7.18, *p* = 0.007. An independent samples t-test and a Chi-square test indicated that perceived scarcity promotes risk aversion when making risky choices.

### 3.3. Psychological Variables and Risky Choices

Five psychological variables, including emotional state, risk attitude, personality, impulsivity, and self-control, were reported. Hierarchical regression analyses revealed that scarcity decreased risk seeking in the choice task (*B* = −0.10, *SE* = 0.05, *p* = 0.046). When emotional state, risk attitude, personality, impulsivity, and self-control were added to the regression model, *R*^2^ increased from 0.019 to 0.207 with *p* = 0.001. The significant *R^2^* change indicates that the scarcity effect may be explained by some of the psychological variables. More specifically, emotional state, personality, and most of the dimensions in impulsivity and self-control had no impact on risky choices. It should be noted that risk attitude (*B* = 0.09, *SE* = 0.03, *p* = 0.001), urgency in impulsivity (*B* = −0.08, *SE* = 0.04, *p* = 0.040), and deliberate action in self-control (*B* = −0.09, *SE* = 0.04, *p* = 0.041) influenced risky choices. However, the results did not reveal any differences in risk attitude (*p* = 0.30), urgency (*p* = 0.43), and deliberate action (*p* = 0.93) between the scarcity condition and the control condition, so the difference in risky choice between the scarcity condition and the control condition was not caused by risk attitude, urgency, or deliberate action.

There were no significant differences between male and female participants in the present study: *t*(211) = 1.45, *p* = 0.15. In both the scarcity and the control condition, no gender differences were observed.

Before the experiment, 24 participants (11.27%) indicated that they had confirmed hypoglycemia. After the experiment, all participants indicated that they did not experience hypoglycemic-like symptoms during the experiment. Risk-taking differences between the scarcity condition and the control condition would not have been affected by ego depletion.

## 4. General Discussion

### 4.1. Conclusions

In the present study, participants in the scarcity condition showed more risk aversion (i.e., a higher proportion chose the safe option) than participants in the control condition. The hypothesis that perceived scarcity induces risk aversion when making a risky choice with a sure gain was supported.

### 4.2. Discussion

A possible reason for the results is that resource scarcity induces individual promotion focus [29]. According to Higgins (1998) and Peng et al. (2016), a promotion focus is concerned with gains, whereas a prevention focus is concerned with losses [29,30]. In a scarcity situation, individuals with promotion focus were inclined to obtain resources so that scarcity would be alleviated. Therefore, when a safe option with a sure gain and other risky options were presented to the participants in the scarcity condition at the same time, selecting the safe option for a sure gain was a better strategy to alleviate their scarcity. According to Haushofer and Fehr (2014), the anticipation of resource scarcity would lead people in the scarcity condition to prefer a safe option over a risky option [31]. And people in the scarcity condition tended to choose immediate and sure benefits and could not delay gratification in exchange for long-term and risky benefits [7]. The impacts of perceived scarcity on individual focus should be tested further. Also, the effect of perceived abundance on risky choices is in need of further consideration, and the effect of perceived scarcity on risky choices without a safe option and with a sure gain needs to be tested in future research.

### 4.3. Contribution

Perceived scarcity in this study was a subjective feeling. This study explored a new possible reason for risk taking and subsequently demonstrated the effect of perceived resource scarcity on risk taking when making risky decisions. It is worth mentioning that money scarcity, absolute or relative, is not the only resource that affects risk taking. Shared resources and common resources were found to increase risky behaviors (e.g., conflict behavior or corruption) [31]. According to Mullainathan and Shafir (2014), people’s cognitive capacity and executive control is reduced when they feel they have less than they would like [1]. Thus, examining the effect of perceived scarcity induced by different resources is important. The conclusions in the present study lay a foundation for future research in this area.

### 4.4. Future Research Direction

Firstly, why and how perceived scarcity affects risk taking should be considered in future research. Perceived scarcity may affect people’s risk perception and the expected benefits, changing their propensity for risk taking. Secondly, existing studies have suggested that scarcity may trigger negative emotions, a sense of insecurity, and a sense of inequality, which were found to increase or decrease risk taking in previous studies. Research has highlighted the possible dimensions or structures of perceived scarcity and negative emotions. A sense of insecurity and a sense of inequality may be the mediation between scarcity and risk taking. Thus, further studies on defining perceived scarcity in different domains and psychological mediating mechanisms are required.

### 4.5. Limitations

This study has several limitations. Firstly, cultural differences were not considered in this study. Some previous studies suggested that people in developing countries were more risk averse than people in developed countries [32]. However, according to Weber and Hsee (2000), people in China showed more risk-seeking behavior than Americans when making financial decisions [33]. This suggests that cultural differences may affect the results and the universality of conclusions in the present study. Secondly, the percentage of female participants in the present study was significantly higher than the percentage of male participants, so the gender differences may be hidden. Thirdly, it should be noted that risky choices in the present study were expressed with positive framing. Future research is needed to investigate the effect of framing on perceived scarcity. Fourthly, we did not control SES in the present study. People at lower SES would be assumed to experience more scarcity, which may affect scarcity perception.

## Figures and Tables

**Table 1:** Descriptive statistics for measures.

Measure		M	SD	Cronbach’s *α*
Perceived scarcity		4.94	0.85	0.667
Emotion	Positive emotion	28.99	5.06	0.855
	Negative emotion	19.37	4.76	0.821
Risk attitude	GRiPS	3.70	1.22	0.941
Personality	Extroversion	4.60	1.33	0.661
	Agreeableness	5.31	0.96	0.102
	Conscientiousness	4.35	1.12	0.518
	Emotional stability	4.61	1.20	0.589
	Openness to experiences	4.46	0.85	0.622
Impulsivity	Premeditation	4.67	0.70	0.815
	Urgency	3.55	0.92	0.880
	Sensation seeking	4.11	1.06	0.867
	Perseverance	4.52	0.63	0.752
Self-control	Self-discipline	4.07	0.56	0.531
	Deliberate action	4.32	0.92	0.830
	Healthy habits	5.05	0.92	0.689
	Work ethics	3.92	0.94	0.647
	Reliability	5.01	0.74	0.492

Note: N = 213.

**Table 2:** The choice task.

Description		Risk of Receiving CNY 0
Safe Option	Receiving CNY 20 (USD 2.86) directly	0.00
Risky Option	80% probability of receiving CNY 25 (USD 0)	0.20
	66% probability of receiving CNY 33 (USD 4.73)	0.40
	40% probability of receiving CNY 50 (USD 7.16)	0.60
	30% probability of receiving CNY 67 (USD 9.59)	0.70
	20% probability of receiving CNY 100 (USD 14.32)	0.80
	10% probability of receiving CNY 200 (USD 28.64)	0.90

## Data Availability

The data presented in this study are available on request from the corresponding author.

## References

[1] Mullainathan S., Shafir E. (2014). Scarcity: Why Having too Little Means so Much.

[2] Liang S.J., Fan P., Yang G.Y. (2021). The effect of perceived scarcity: Experiencing scarcity increases risk taking. J. Psychol..

[3] Shah A.K., Mullainathan S., Shafir E. (2012). Some Consequences of Having Too Little. Science.

[4] Huijsmans I., Ma I., Micheli L., Civai C., Stallen M., Sanfey A.G. (2019). A scarcity mindset alters neural processing underlying consumer decision making. Proc. Natl. Acad. Sci. USA.

[5] Williams C.C., Saffer B.Y., Mcculloch R.B., Krigolson O.E. (2016). The scarcity heuristic impacts reward processing within the medial-frontal cortex. Neuro Rep..

[6] Kaplan H.S., Gangestad S.W. (2005). Handbook of Evolutionary Psychology.

[7] Griskevicius V., Tybur J.M., Delton A.W., Robertson T.E. (2011). The influence of mortality and socioeconomic status on risk and delayed rewards: A life history theory approach. J. Personal. Soc. Psychol..

[8] Griskevicius V., Ackerman J.M., Cantu S.M., Delton A.W., Robertson T.E., Simpson J.A., Thompson M.E., Tybur J.M. (2013). When the Economy Falters, Do People Spend or Save? Responses to Resource Scarcity Depend on Childhood Environments. Psychol. Sci..

[9] Caraco T., Martindale S., Whittam S.T. (1980). An empirical demonstration of risk-sensitive foraging preferences. Anim. Behav..

[10] Mishra S., Fiddick L. (2012). Beyond gains and losses: The effect of need on risky choice in framed decisions. J. Personal. Soc. Psychol..

[11] Mishra S., Lalumière M.L. (2010). You can’t always get what you want: The motivational effect of need on risk-sensitive decision-making. J. Exp. Soc. Psychol..

[12] Pietras C.J., Hackenberc T.D. (2001). Risk-sensitive choice in humans as a function of an earnings budget. J. Exp. Anal. Behav..

[13] Gonzales J., Mishra S., Camp R.D. (2016). For the win: Risk-sensitive decision-making in teams. J. Behav. Decis. Mak..

[14] Song Y., Xu R., Xing C. (2017). Risk-sensitivity theory: Need motivates risky decision-making. Adv. Psychol. Sci..

[15] Payne B.K., Brown-Iannuzzi J.B., Hannay J.W. (2017). Economic inequality increases risk taking. Proc. Natl. Acad. Sci. USA.

[16] Kugler T., Connolly T., Ordonez L.D. (2012). Emotion, Decision, and Risk: Betting on Gambles versus Betting on People. J. Behav. Decis. Mak..

[17] Soane E., Dewberry C., Narendran S. (2010). The role of perceived costs and perceived benefits in the relationship between personality and risk-related choices. J. Risk. Res..

[18] Mishra S., Barclay P., Sparks A.M. (2017). The Relative State Model Integrating Need-Based and Ability-Based Pathways to Risk-Taking. Personal. Soc. Psychol. Rev..

[19] Dou K., Nie Y., Wang Y., Li J. (2014). Ego depletion promotes risk- taking behavior. J. Psychol. Sci..

[20] Roux C., Goldsmith K., Bonezzi A. (2015). On the psychology of scarcity: When reminders of resource scarcity promote selfish (and generous) behavior. J. Consum. Res..

[21] Mishra S., Hing L.S., Lalumiere M.L. (2015). Inequality and Risk-Taking. Evol. Psychol..

[22] Clark L.A., Watson D. (1988). Mood and the mundane: Relations between daily life events and self-reported mood. J. Personal. Soc. Psychol..

[23] Huang L., Yang T., Ji Z. (2003). Applicability of the Positive and the Negative Affect Scale in China. Chin. Ment. Health J..

[24] Zhang D.C., Highhouse S., Nye C.D. (2019). Development and validation of the General Risk Propensity Scale (GRiPS). J. Behav. Decis. Mak..

[25] Gosling S.D., Rentfrow P.J., Swann W.B. (2003). A very brief measure of the Big-Five personality domains. J. Res. Personal..

[26] Whiteside S.P., Lynam D.R. (2001). The Five Factor Model and impulsivity: Using a structural model of personality to understand impulsivity. Personal. Individ. Differ..

[27] Tangney J.P., Baumeister R.F., Boone A.L. (2004). High Self-control Predicts Good Adjustment, Less Pathology, Better Grades, and Interpersonal Success. J. Pers..

[28] Unger A., Bi C., Xiao Y.Y., Ybarra O. (2016). The revising of the Tangney Self-Control Scale for Chinese students. Psych J..

[29] Peng Y., Wang X., Wu S., Jin S., Sun R. (2016). A brief introduction of Life History Theory and its combination with social psychology: Moral behaviors as an example. Adv. Psychol. Sci..

[30] Higgins E.T. (1998). Promotion and Prevention: Regulatory Focus as A Motivational Principle. Adv. Exp. Soc. Psychol..

[31] Haushofer J., Fehr E. (2014). On the psychology of poverty. Science.

[32] Allen M.W., Bettinger R.L., Codding B.F. (2016). Resource scarcity drives lethal aggression among prehistoric hunter-gatherers in central California. Proc. Natl. Acad. Sci. USA.

[33] Weber E., Hsee C. (2000). Culture and individual judgment and decision making. Appl. Psychol..

